# Addendum: Manabu, K., et al. Prognostic Significance of Aberrant Claudin-6 Expression in Endometrial Cancer. *Cancers* 2020, *12*, 2748

**DOI:** 10.3390/cancers13050971

**Published:** 2021-02-26

**Authors:** Manabu Kojima, Kotaro Sugimoto, Mizuko Tanaka, Yuta Endo, Hitomi Kato, Tsuyoshi Honda, Shigenori Furukawa, Hiroshi Nishiyama, Takafumi Watanabe, Shu Soeda, Keiya Fujimori, Hideki Chiba

**Affiliations:** 1 Department of Basic Pathology, Fukushima Medical University School of Medicine, Fukushima 960-1295, Japan; m2149@fmu.ac.jp (M.K.); mizuko@fmu.ac.jp (M.T.); hiace_tokotsu@yahoo.co.jp (Y.E.); m151037@fmu.ac.jp (H.K.); 2 Department of Obstetrics and Gynecology, Fukushima Medical University School of Medicine, Fukushima 960-1295, Japan; s-furu@infoseek.jp (S.F.); wata@fmu.ac.jp (T.W.); s-soeda@fmu.ac.jp (S.S.); fujimori@fmu.ac.jp (K.F.); 3 Department of Obstetrics and Gynecology, Iwaki City Medical Center, Iwaki 973-8402, Japan; boo.honda@gmail.com (T.H.); nishi-ya@fmu.ac.jp (H.N.)

The authors wish to make the following correction to this paper [1].

The authors would like to acknowledge Yuko Hashimoto and Shigeyuki Asano for providing cancer tissues. Therefore, the following sentence should be added in the “Acknowledgments” section:

“We are also grateful to Yuko Hashimoto and Shigeyuki Asano for providing cancer tissues.”

The authors would like to apologize for this omission from the original manuscript. The change does not affect the scientific results.
