# Supplementary material for: Prognostic Significance of Aberrant Claudin-6 Expression in Endometrial Cancer
Source: Cancers (Basel). 2020 Sep 24;12(10):2748. doi: 10.3390/cancers12102748 (PMC7656298; doi:10.3390/cancers12102748)
Supplement: Supplementary file 1 [file cancers-12-02748-s001.zip › cancers-932032-supplementary-send conversion.pdf]

cancers

Supplementary Materials

Prognostic Significance of Aberrant Claudin-6 Expression in Endometrial Cancer

Manabu Kojima, Kotaro Sugimoto, Mizuko Tanaka, Yuta Endo, Hitomi Kato, Tsuyoshi Honda, Shigenori Furukawa, Hiroshi Nishiyama, Takafumi Watanabe, Shu Soeda, Keiya Fujimori and Hideki Chiba

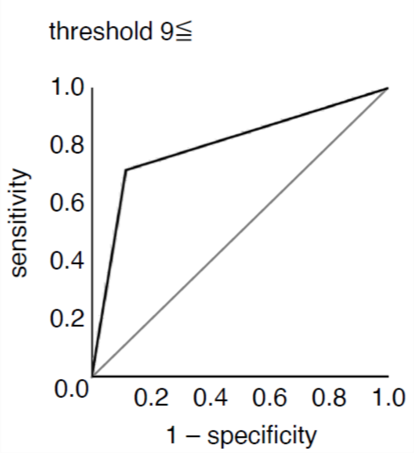

Figure S1. The receiver operating characteristic (ROC) curves for overall survival using a cutoff score of IRS≧8. The reference line is shown in gray.
