# Supplementary figures and images for: Prognostic Significance of Aberrant Claudin-6 Expression in Endometrial Cancer
Source: Cancers (Basel). 2020 Sep 24;12(10):2748. doi: 10.3390/cancers12102748 (PMC7656298; doi:10.3390/cancers12102748)

Uncropped and unadjusted gel images

Figure. 1C

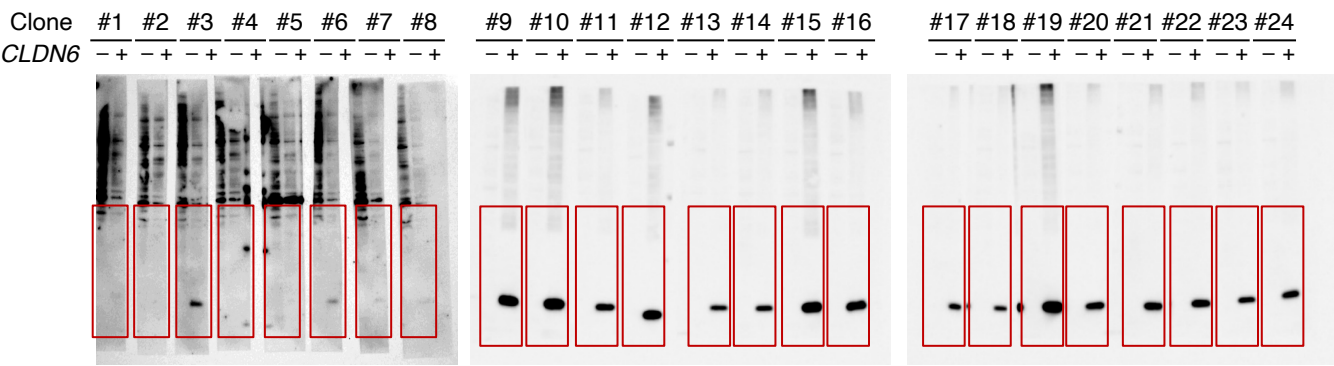

Figure. 1D

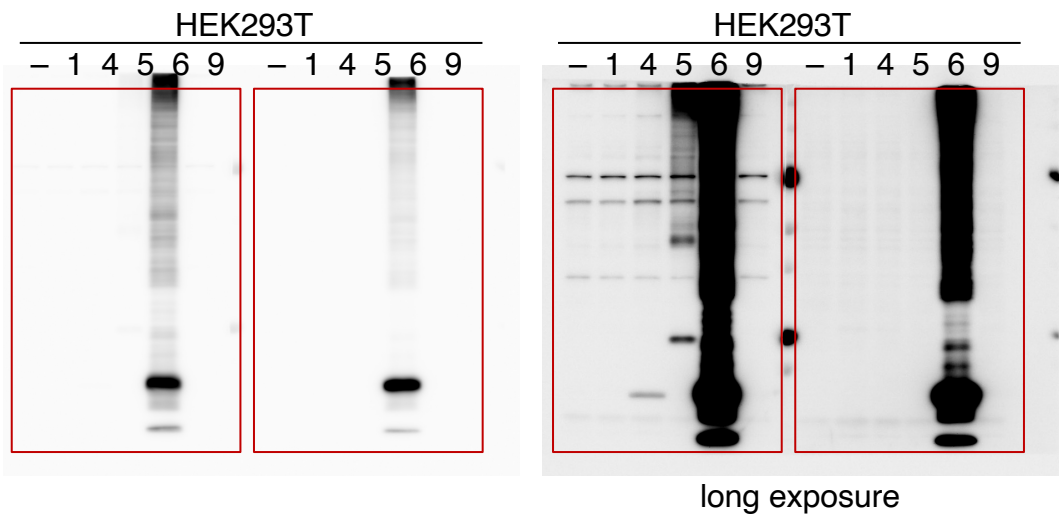

Supplement: Supplementary file 1 [file cancers-12-02748-s001.zip › cancers-932032-original images.pdf]
